# Incidence rate of psychiatric disorders in 2020: The pivotal role played by SARS-CoV-2 infection

**DOI:** 10.1371/journal.pone.0274330

**Published:** 2022-09-22

**Authors:** Antonio L. Teixeira, Regina M. Hansen, Joseph S. Wozny, Caroline M. Schaefer, Rodrigo Machado-Vieira, Lokesh Shahani, Scott D. Lane, Jair C. Soares, Trudy M. Krause

**Affiliations:** 1 Department of Psychiatry and Behavioral Sciences, McGovern Medical, The University of Texas Health Science Center at Houston, Houston, TX, United States of America; 2 School of Public Health, The University of Texas Health Science Center at Houston, Houston, TX, United States of America; Yale School of Medicine, UNITED STATES

## Abstract

**Importance:**

The Coronavirus Disease (COVID-19) pandemic has significantly impacted mental health outcomes. While the frequency of anxiety and depressive symptoms has increased in the whole population, the relationship between COVID-19 and new psychiatric diagnoses remains unclear.

**Objective:**

To compare the population incidence rate of emergence of *de novo* psychiatric disorders in 2020 compared to the previous years, and to compare the incidence rate of new psychiatric disorder diagnoses between people with vs without COVID-19.

**Design, setting, and participants:**

This study utilized administrative claims data from the Clinformatics® Data Mart database, licensed from Optum®. The study is a cross-sectional analysis that compared the incidence rate of new psychiatric disorders in 2020 vs. 2018 and 2019 in the entire insured population database. Subsequently, the incidence of new psychiatric disorders in people with vs. without COVID-19 during 2020 was analyzed.

**Exposure:**

The exposures included diagnosis and severity of COVID-19 infection.

**Main outcomes measures:**

The dependent variables of interest were the incidence rates of new psychiatric disorders, specifically schizophrenia spectrum disorders, mood disorders, anxiety disorders, and obsessive-compulsive disorder.

**Results:**

The population studied included 10,463,672 US adults (mean age 52.83, 52% female) who were unique people for the year of 2020. Incidence of newly diagnosed psychiatric disorders per 1,000 individuals in the 2020 whole population were 28.81 (CI: 28.71, 28.92) for anxiety disorders, 1.04 (CI: 1.02, 1.06) for schizophrenia disorders, 0.42 (CI: 0.41, 0.43) for OCD and 28.85 (CI: 28.75, 28.95) for mood disorders. These rates were not significantly higher than 2018 or 2019. When comparing incidence rates between COVID-19 vs. non-COVID-19 populations in 2020, the rates were significantly higher in the COVID-19 population: 46.89 (CI: 46.24, 47.53) for anxiety, 49.31 (CI: 48.66, 49.97) for mood disorders, 0.57 (CI: 0.50, 0.65) for OCD, and 3.52 (CI: 3.34, 3.70) for schizophrenia. COVID-19 severity was significantly associated with new diagnoses of schizophrenia, anxiety and mood disorders in multivariate analyses.

**Conclusions:**

Compared to 2018 and 2019, in 2020 there was no increased incidence of new psychiatric disorders in the general population based on insurance claims data. Importantly, people with COVID-19 were more likely to be diagnosed with a new psychiatric disorder, most notably disorders with psychosis, indicating a potential association between COVID-19 and mental/brain health.

## Introduction

The year of 2020 was marked by the Coronavirus Disease (COVID-19) pandemic. To ‘flatten the curve’ of infection and, therefore, minimize morbidity/mortality, several public health measures were imposed to limit social interactions, resulting in both interpersonal isolation and economic loss [1, 2]. Difficulties around the length and impact of the pandemic, extent of socioeconomic changes, and conflicting messages from public authorities contributed to widespread emotional stress and increased risk for mental health problems [2–4]. Previous studies have consistently implicated stress as a trigger of psychiatric disorders, including anxiety, mood, and psychotic disorders [5].

Despite awareness of the potential impact of COVID-19 pandemic on mental health since the early phases of the pandemic [6, 7], public health parameters have not been fully determined with regard to onset of documented psychiatric disorders. Given the lessons from previous viral pandemics, such as the Severe Acute Respiratory Syndrome (SARS) in 2002–2003, the impact is expected to be significant [8]. In the first months of the pandemic (up to May 2020), symptoms of anxiety and depression were increased in the general population compared to pre-pandemic levels, with pre-existing psychiatric conditions, female sex, and concerns about getting infected consistently reported as risk factors [9]. In the United States (US), The Household Pulse Survey, an experimental data system run by the National Center for Health Statistics (NCHS) partnered with the Census Bureau, reported that symptoms of anxiety and depression increased considerably during April–July and August–October of 2020 compared with the same periods in 2019 [10]. A web-based survey found that the prevalence of anxiety disorders, defined as score ≥3 out of 6 on the Generalized Anxiety Disorder-2, was three times those reported in the second quarter of 2019 (25.5% versus 8.1%), and the prevalence of depressive disorder, defined as score ≥3 out of 6 on the Patient Health Questionnaire-4, was approximately four times that reported in the second quarter of 2019 (24.3% versus 6.5%) [11]. A telephone-based survey with 1,470 US residents aged 18 years or older confirmed that prevalence of depression symptoms was more than 3-fold higher during COVID-19 compared with before the pandemic, and subjects with lower economic and social resources, and greater exposure to stressors (e.g., job loss) had a greater burden of these symptoms [12]. Note that these studies measured self-reported psychiatric symptoms rather than medically documented psychiatric disorders.

In addition to increased frequency of psychiatric symptoms at the societal level, data also suggested that people infected with the severe acute respiratory syndrome coronavirus 2 (SARS-CoV-2) developed neuropsychiatric symptoms at a higher rate than those without COVID-19 [13, 14]. SARS-CoV-2 has been directly and indirectly implicated in brain dysfunction associated with both acute and persistent/chronic neuropsychiatric symptoms and disorders [13–16]. In a high percentage of cases, the so-called ‘long-haulers’ or ‘long COVID’, neuropsychiatric symptoms developed and/or persisted well after the resolution of the acute illness [16, 17]. Long COVID is a complex and heterogeneous syndrome comprising over 60 physical and psychological symptoms, such as malaise, fatigue, dyspnea, pain, impaired concentration, anxiety, depression, among others [18–20]. In an electronic health records (EHR) study with 236,379 patients diagnosed with COVID-19 the estimated incidence of a neurological or psychiatric diagnoses in the following 6 months was over 32%, with 12·8% receiving their first such diagnosis [21]. The risks of development of such conditions were greater in patients who had severe COVID-19 (21). The collective evidence suggests that a major consequence of the COVID-19 pandemic would be a mental health crisis. Moreover, patients with major psychiatric disorders would have worse clinical outcomes related to COVID-19 [22]. Several studies and related meta-analyses have confirmed this mental health crisis, but recent studies also present a complex picture, calling for further and more granular research [9].

Leveraging access to the Optum® national claims data, a large administrative database representing over 27 million persons annually, we aimed to investigate the mental health impact of COVID-19 in 2020. Claims data provide a comprehensive history of individuals’ diagnoses and treatment, as all services documented over time regardless of provider or provider system. Moreover, claims data allow for the investigation medically-documented psychiatric disorders rather than the presence of psychiatric symptoms. The specific objectives of this study were: (1) to compare the population incidence rate of major psychiatric disorders in 2020 compared to the previous two years (2018–2019); and (2) to compare the incidence rate of new psychiatric disorder diagnoses between people with vs. without COVID-19. Our hypotheses were (1) that the incidence rates of major psychiatric diagnoses in the whole population would be higher in 2020 compared to 2018 and 2019, and (2) people infected with COVID-19 would be diagnosed with new psychiatric disorders at higher rates than people without COVID-19. Lastly, we explored, based on an ordinal ranking, if severity of COVID-19 was associated with increased likelihood of a diagnosed psychiatric disorder.

## Methods

### Study design and data collection

This is a cross-sectional study based on Optum’s Clinformatics® Data Mart (CDM), which is derived from a database of administrative health claims for members of large commercial and Medicare Advantage health plans. The data represent over 27 million persons annually in the US. Available data include demographics, medical and psychiatric diagnoses (ICD-10 codes), coded and dated procedures, and resource utilization (e.g. hospital admission, non-invasive and invasive ventilation support, etc). All relevant data are within the manuscript. Data used for this study was obtained by a licensed agreement from Optum. For information regarding licensing Optum data assets, other researchers may contact Optum Clinformatics Data Mart at https://www.optum.com/business/contact.html#sales, where there is a contact form to request information on purchase and access to data or analytics.

The study (HSC-SPH-20-0758) was approved by the institutional review board of The University of Texas Health Science Center at Houston with a waiver of authorization for informed consent based on exempt status according to 45 CFR 46.101(b), with the de-identification of personal health information from the Optum database, and the retrospective observational study design. This study adheres to the STROBE reporting guideline [23].

### Population and definitions

**Fig 1:**
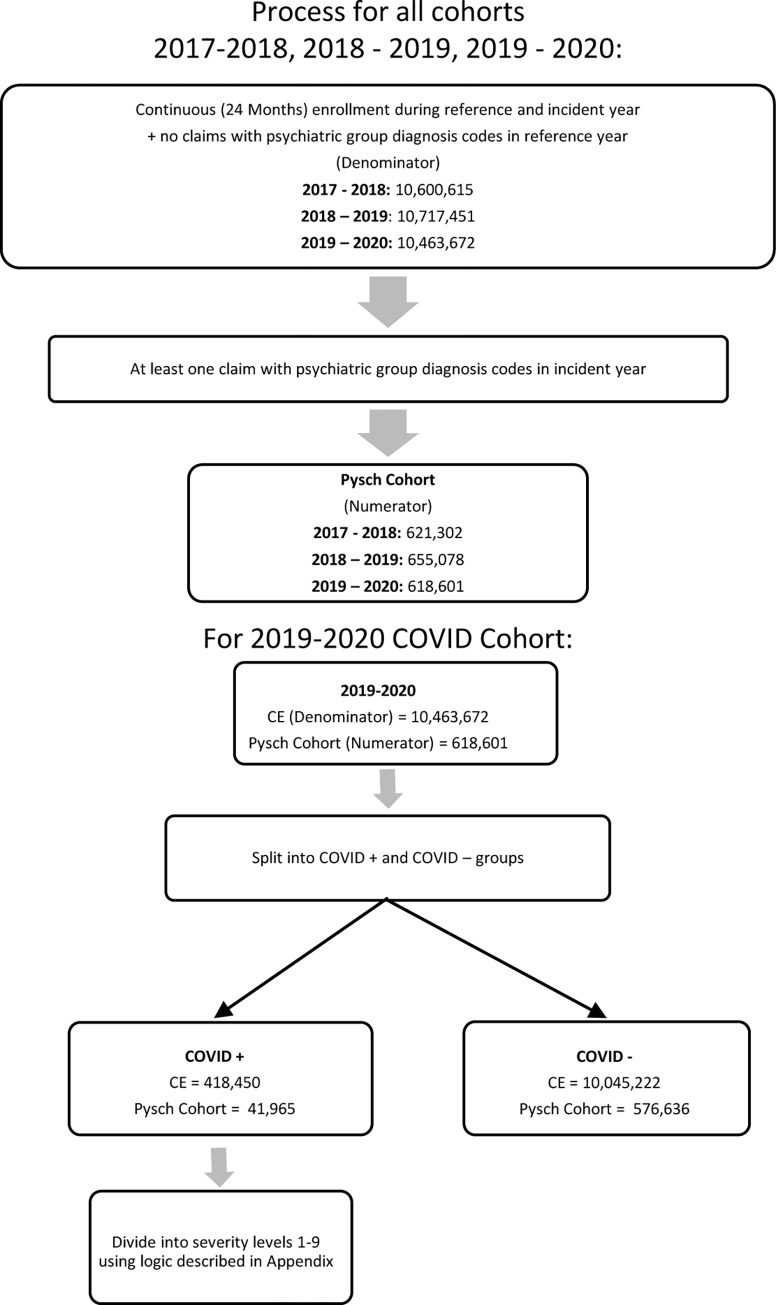
Inclusion flow chart.

The study groups consisted of persons of all ages with continuous enrollment in 2017–2018, 2018–2019, and 2019–2020. Claim history was then searched to exclude persons with a psychiatric diagnosis in the prior year in order to identify newly diagnosed psychiatric disorders in 2018, 2019 and 2020, and allowing for one full prior year with no psychiatric diagnosis. Only the diagnoses of interest satisfied the exclusion criteria. Thus, we started with a total population count of 10,600,615 (52% female) in 2018, 10,717,451 in 2019 (52% female) and 10,463,672 (53% female) in 2020. Of those in 2020, 418,450 had a diagnosis of COVID-19 (4.00%, CI: (3.99, 4.01). Patients were placed into the general population and emergent cohort using the following logic and the diagnosis codes described below it (Fig 1).

Using International Statistical Classification of Diseases (ICD) diagnosis codes, each cohort was split into four groups of diagnoses: (1) schizophrenia spectrum disorders (ICD codes: F20x, F22x, F23x, F25x, F28, F29), (2) mood disorders (F30x, F31x, F32x, F33x F34x, F39x, (3) anxiety disorders (F40x, F41x), (4) obsessive-compulsive disorders (F42x). If a patient met criteria for multiple groups, they were placed in only one group for purposes of the study. Assignment to the primary group was based on the following ranking, according to DSM-5 hierarchical diagnosis: (1) schizophrenia spectrum disorders, (2) mood disorders, (3) obsessive-compulsive disorders, (4) anxiety disorders.

Patients within the positive group of COVID-19 were placed into groups based upon the severity of their condition. The severity scale was designed specifically for use with claims data by the researcher and was based on the COVID-19 Progression Scale of the World Health Organization [24]. The claims severity scale relies on diagnoses in claims with confirmed COVID-19, also allowing self-reported COVID-19. The level 1 was noted as unconfirmed report of COVID-19, and was created to allow for studies assessing post-COVID-19 effects. The severity scale ranges from level 1 (unconfirmed) to level 9 (death in hospital). Death due to COVID-19 could only be confirmed if the death occurred in a hospital or during treatment. The severity levels were assigned based upon diagnoses and procedure codes from the claims (see S1 Appendix for level assignment details).

### Covariates and confounders

In order to compare the 2020 population that had COVID-19 and those that did not have COVID-19 and its impact on each of the emergent conditions, we identified the following covariates for each patient: age, race/ethnicity, sex, alcohol use and smoking status, and a count of comorbidities including hypertension, diabetes, chronic kidney disease, coronary artery disease, ischemic heart disease, metabolic syndrome, and chronic obstructive pulmonary disease. The number of comorbidities was identified according to the ICD-10 codes in the Elixhauser Comorbidity Index [25]. Psychiatric diagnoses in the index were excluded from the count of comorbidities because they are outcomes for the study. Alcohol and nicotine were excluded from the comorbidity count because they are covariates. Race and ethnicity were determined by patient report and were included due to established differences in severity of COVID-19 [26].

### Outcome measures

The incidence rate of new diagnoses of psychiatric disorders (i.e. Schizophrenia, Mood Disorders, Anxiety Disorders, and Obsessive-Compulsive Disorders) was determined for the members with continuous enrollment in all years for each cohort: 2017–2018, 2018–2019 and 2019–2020. The outcomes of comparison were annual incidence rate of emergent psychiatric disorders. Later, the incidence rate in 2020, along with demographic and clinical variables, were compared between the subset of the overall population who contracted COVID-19 in 2020 versus the remaining subpopulation that did not contract COVID-19 in 2020. Further analysis in 2020 was conducted to evaluate the impact of the severity of COVID-19 on the incidence rates while controlling for age, gender and race/ethnicity.

### Statistical analysis

The statistical results were generated using Version 9.4 of the SAS System (SAS Institute Inc., Cary, NC, USA). Incidence rates per 1,000 individuals of newly diagnosed psychiatric disorders were identified for each year from 2018 to 2020. Differences in incidence of new psychiatric diagnosis between years were determined with a difference analysis using logistic regressions for each psychiatric diagnosis as the outcome and predictor variables including a control group indicating which set of years was being compared, year, and an interaction variable consisting of the control group and year. The interaction terms were used to determine if the change between years was significant.

After comparing the differences in the emergence of new psychiatric diagnoses across the years, the analysis focused on 2020. First, a bivariate analysis was performed comparing patients with and without COVID-19. Counts and percentages of age, gender, race/ethnicity, number of comorbidities, alcohol or nicotine use and incidence of various psychiatric disorders were compared across both groups and assessed using a chi-square test.

A logistic regression was implemented for each psychiatric disorder in those with a COVID-19 diagnosis to determine the role played by the severity of the infection. Severity was the primary predictor, while controlling for age, gender and race/ethnicity. Severity level 2 was used as the reference level as that was the lowest severity level of a confirmed COVID-19 diagnosis. Because the count of OCD COVID-19 patients was low, only a small number reached the more extreme severity levels. Therefore to maintain integrity in the regression, the severity levels in the OCD model were regrouped into 3 combined levels representing ambulatory (levels 1–3), emergency room or hospitalization without organ support (levels 4–6), and hospitalization with organ support or death (levels 7–9).

## Results

This study included three cohorts characterized by year from 2018 to 2020 (Fig 1, S1 Table). The population count stayed relatively consistent across the years consisting of 10,600,615 individuals in 2018, 10,717,451 in 2019 and 10,463672 in 2020 (Fig 1). Across all years, those over 55 years old made up the majority of the population (> 51%, S1 Table). White individuals made up more than 70% and females more than 50% of the cohort for each year (S1 Table). The proportion of those with one or more comorbidities went up slightly while alcohol and nicotine use decreased each year (S1 Table).

When comparing rates of new psychiatric diagnoses across years, incidence rates per 1,000 individuals of OCD remained constant, and there was a decrease in schizophrenia and mood disorders in 2020 (Table 1). The incidence rate per 1,000 individuals of those with anxiety increased in 2020 (28.81, CI: 28.71, 28.92) compared to 2019 (28.29, CI: 28.19, 28.39). However, when assessing change in differences, the significant time interaction (P<0.0001) indicated a significant decrease in the rates of anxiety from 2019 to 2020. Therefore, we observed a change in anxiety that was lower than trends observed in prior years.

**Table 1:** Incidence rate per 1,000 people and trends of new psychiatric disorders between 2018 and 2020.

Characteristic	2018		2019		2020		2018–2019	2019–2020	Change in rates
	Rate	95% CI	Rate	95% CI	Rate	95% CI	Diff 95% CI	Diff 95% CI	p-value
**Anxiety**	27.03	26.93, 27.13	28.29	28.19, 28.39	28.81	28.71, 28.92	-1.40, -1.13	-0.66, -0.38*	<0.0001
**Mood**	30.06	29.95, 30.16	31.27	31.17, 31.38	28.85	28.75, 28.95	-1.36, -1.07	2.28, 2.57*	<0.0001
**Schizophrenia**	1.15	1.13, 1.17	1.15	1.13, 1.17	1.04	1.02, 1.06	-0.03, 0.03	0.09, 0.14*	<0.0001
**OCD**	0.37	0.36, 0.38	0.40	0.39, 0.42	0.42	0.41, 0.43	-0.05, -0.01	-0.03, 0.00	0.7179

*significance in the rate of change from year to year

Note: Change in rates p-values represent time interaction terms of respective models

Considering only the year 2020, first we compared sociodemographic and clinical parameters between people with vs. without COVID-19 (Table 2). The bivariate analysis revealed statistically significant differences amongst demographics and comorbidities in terms of COVID-19. Females (COVID-19: 54.25% vs. No COVID-19: 52.39%), Black (COVID-19: 11.56% vs. No COVID-19: 10.21%), and Hispanic individuals (COVID-19: 17.62% vs. No COVID-19: 12.90) were more likely to have a COVID-19 diagnosis as well as those with at least one comorbidity (COVID-19: 78.27% vs. No COVID-19: 65.14%). There were also higher percentages of individuals with alcohol (COVID-19: 2.91% vs. No COVID-19: 2.16%), or nicotine dependence (COVID-19: 8.67% vs. No COVID-19: 8.07%) in those with COVID-19.

**Table 2:** Comparison of demographics and new psychiatric disorders between people with COVID-19 and those without COVID-19 in 2020.

	COVID-19			
Characteristic	Yes	95% CI	No	95% CI
**Age Group**	n (%)		n (%)	
0–19	31,122 (7.44)	7.36, 7.52	1,341,445 (13.35)	13.33, 13.38
20–34	59,166 (14.14)	14.03, 14.24	1,205,166 (12.00)	11.98, 12.02
35–44	46,822 (11.19)	11.09, 11.28	1,023,105 (10.18)	10.17, 10.20
45–54	54,633 (13.06)	12.95, 13.16	1,075,358 (10.71)	10.69, 10.72
55–64	58,518 (13.98)	13.88, 14.09	1,236,366 (12.31)	12.29, 12.33
65–74	77,251 (18.46)	18.34, 18.58	2,117,507 (21.08)	21.05, 21.11
75 +	90,938 (21.73)	21.61, 21.86	2,046,275 (20.37)	20.35, 20.40
**Gender**				
Female	226,997 (54.25)	54.10, 54.40	5,263,139 (52.39)	52.36, 52.43
Male	191,453 (45.75)	45.60, 45.90	4,782,083 (47.61)	47.57, 47.64
**Race/Ethnicity**				
White	262,042 (67.74)	67.59, 67.88	6,504,572 (71.56)	71.53, 71.59
Black	44,734 (11.56)	11.46, 11.66	927,985 (10.21)	10.19, 10.23
Asian	11,924 (3.08)	3.03, 3.14	485,067 (5.34)	5.32, 5.35
Hispanic	68,162 (17.62)	17.50, 17.74	1,172,521 (12.90)	12.88, 12.92
**One or more Comorbidity**	327,539 (78.27)	78.15, 78.40	6,543,219 (65.14)	65.11, 65.17
**Alcohol Use**	12,158 (2.91)	2.85, 2.96	216,866 (2.16)	2.15, 2.17
**Nicotine Use**	36,292 (8.67)	8.58, 8.76	810,576 (8.07)	8.05, 8.09
**n (Incidence Rate by 1000)**				
Anxiety	19,619 (46.89)	46.24, 47.53	281,885 (28.06)	27.96, 28.10
Mood	20,635 (49.31)	48.66, 49.97	281,218 (28.00)	27.89, 28.10
Schizophrenia	1,471 (3.52)	3.34, 3.70	9,404 (0.94)	0.92, 0.96
OCD	240 (0.57)	0.50, 0.65	4129 (0.41)	0.40, 0.42

Notes: all p-values <0.0001, chi-square tests.

Differences in the rates of new psychiatric diagnoses were evident between people with vs. without COVID-19 (Table 2). Those without COVID-19 had statistically significant lower incidence rates per 1,000 individuals of new diagnoses of anxiety (28.06, CI: 27.96, 28.10), mood disorders (28.00, CI: 27.89, 28.10), schizophrenia (0.94, CI: 0.92, 0.96) and OCD (0.41, CI: 0.40, 0.42) compared to the COVID-19 population: 46.89 (CI: 46.24, 47.53) for anxiety, 49.31 (CI: 48.66, 49.97) for mood disorders, 3.52 (CI: 3.34, 3.70) for schizophrenia, and 0.57 (CI: 0.50, 0.65) for OCD.

**Fig 2:**
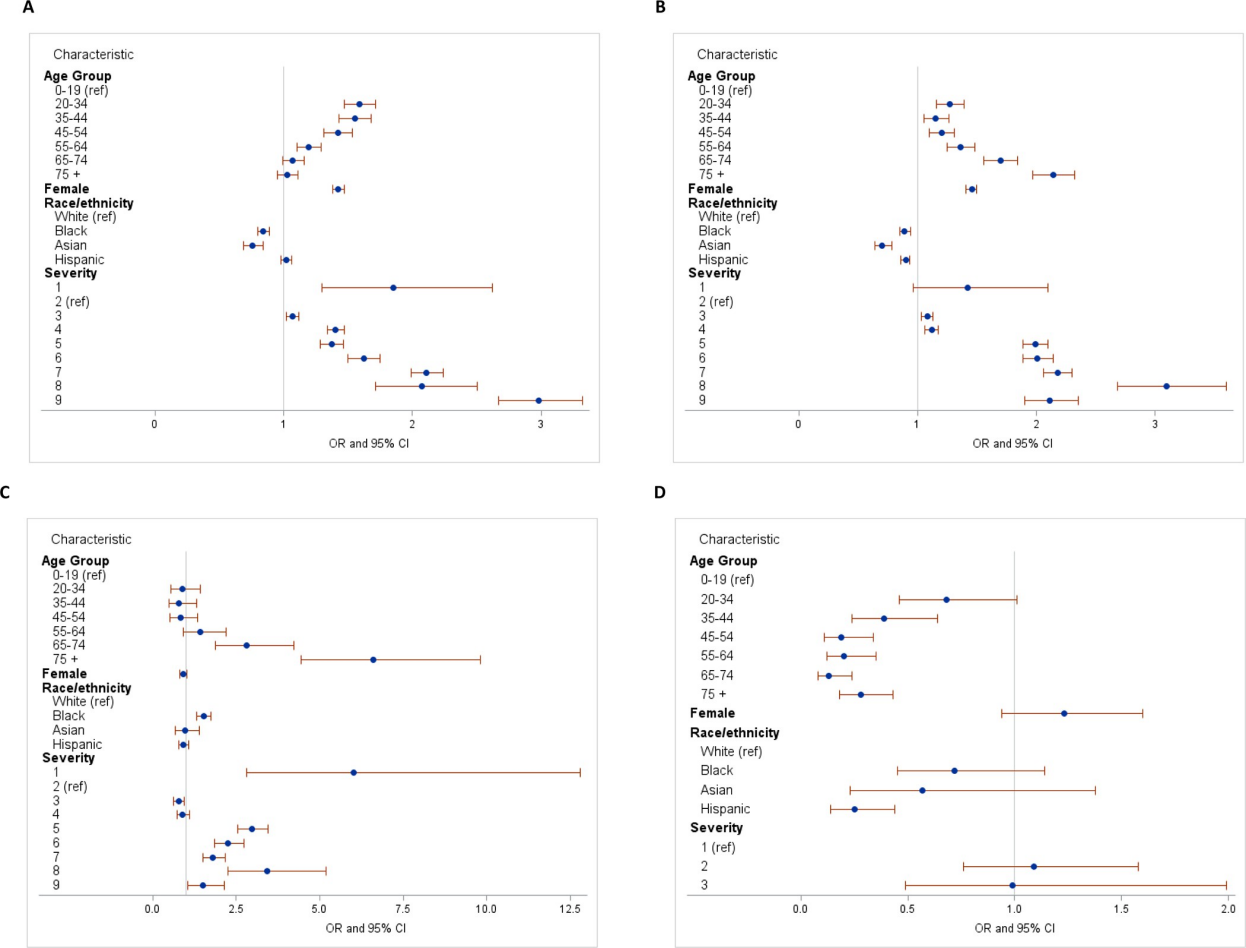
Forest plots of new psychiatric diagnoses and COVID-19 severity controlling for age, gender and race/ethnicity: a) anxiety disorders, b) mood disorders, c) schizophrenia, d) obsessive-compulsive disorder.

When assessing the role of COVID-19 severity on new psychiatric diagnoses, there was a large increase in the odds of developing anxiety in all higher severity levels when controlling for age, gender and race/ethnicity (Table 3; Fig 2). A logistic regression described this positive increasing relationship of anxiety and COVID-19 severity, with odds ratios of 1.07 (CI: 1.02, 1.12) in level 3, 1.62 (CI: 1.50, 1.75) in the midpoint level 6, and 2.98 (CI: 2.67, 3.32) in the most severe level 9, in reference to the least severe cases (Fig 2A). This trend was also observed for mood disorders while controlling for the same demographics, starting at severity level 3 (OR: 1.08, CI: 1.03, 1.13) and peaking at level 8 (OR: 3.10, CI: 2.68, 3.60) (Fig 2B). The schizophrenia model showed a marked difference in having a newly diagnosed schizophrenia spectrum disorder at higher levels of severity while controlling for age, gender and race/ethnicity (Fig 2C). Individuals in severity level 5 had 2.96 (CI: 2.55, 3.44) times the odds than in the least severe level, with similar trends seen in level 6 (OR: 2.25, CI: 1.85, 2.73). There was a marked increase in severity level 8 compared to those with less severe cases, with an odds ratio of 3.42 (CI: 2.26, 5.19). There were no significant associations between COVID-19 severity and OCD while controlling for demographics (Fig 2D).

**Table 3:** Logistic regression of new psychiatric diagnoses and COVID-19 severity controlling for age, gender and race/ethnicity.

Characteristic	Anxiety Disorder	Mood Disorder	Schizophrenia	OCD
	OR (95% CI)	OR (95% CI)	OR (95% CI)	OR (95% CI)
Age Group				
0–19	ref	ref	ref	ref
20–34	1.59 (1.47, 1.71)	1.27 (1.16, 1.39)	0.88 (0.55, 1.43)	0.68 (0.46, 1.01)
35–44	1.55 (1.43, 1.68)	1.15 (1.05, 1.26)	0.79 (0.48, 1.31)	0.39 (0.24, 0.64)
45–54	1.42 (1.31, 1.53)	1.20 (1.10, 1.31)	0.84 (0.52, 1.35)	0.19 (0.11, 0.34)
55–64	1.19 (1.10, 1.29)	1.36 (1.25, 1.48)	1.41 (0.91, 2.19)	0.20 (0.12, 0.35)
65–74	1.07 (0.99, 1.16)	1.70 (1.56, 1.84)	2.81 (1.87, 4.22)	0.13 (0.08, 0.24)
75 +	1.03 (0.95, 1.11)	2.14 (1.97, 2.32)	6.60 (4.44, 9.82)	0.28 (0.18, 0.43)
Female	1.42 (1.38, 1.47)	1.46 (1.41, 1.50)	0.92 (0.82, 1.02)	1.23 (0.94, 1.60)
Race/ethnicity				
White	ref	ref	ref	ref
Black	0.84 (0.80, 0.89)	0.89 (0.85, 0.94)	1.52 (1.32, 1.75)	0.72 (0.45, 1.14)
Asian	0.76 (0.69, 0.84)	0.70 (0.64, 0.78)	0.98 (0.68, 1.40)	0.57 (0.23, 1.38)
Hispanic	1.02 (0.98, 1.06)	0.90 (0.86, 0.93)	0.91 (0.77, 1.07)	0.25 (0.14, 0.44)
Severity*				
1	1.85 (1.30, 2.62)	1.42 (0.96, 2.10)	6.01 (2.82, 12.80)	ref
2	ref	ref	ref	
3	1.07 (1.02, 1.12)	1.08 (1.03, 1.13)	0.77 (0.62, 0.95)	
4	1.40 (1.34, 1.47)	1.12 (1.06, 1.17)	0.89 (0.72, 1.10)	1.09 (0.76, 1.58)
5	1.37 (1.28, 1.46)	1.99 (1.89, 2.10)	2.96 (2.55, 3.44)	
6	1.62 (1.50, 1.75)	2.01 (1.89, 2.14)	2.25 (1.85, 2.73)	
7	2.11 (1.99, 2.24)	2.18 (2.06, 2.30)	1.80 (1.49, 2.16)	0.99 (0.49, 1.99)
8	2.07 (1.71, 2.50)	3.10 (2.68, 3.60)	3.42 (2.26, 5.19)	
9	2.98 (2.67, 3.32)	2.11 (1.90, 2.35)	1.49 (1.04, 2.13)	

*The scale of COVID-19 severity is presented in the S1 Appendix.

## Discussion

Based on a large administrative claims database and focused on diagnoses instead of symptoms, our results showed that (1) the rate of new psychiatric disorders did not increase in the whole population in 2020 compared to the previous two years, but (2) the new diagnosis rate markedly increased in people with (vs without) COVID-19 in 2020. In the group with COVID-19, across different psychiatric disorders assessed, the increase was most pronounced in schizophrenia spectrum disorders.

Our results did not confirm the hypothesis that the incidence rate of psychiatric disorders would increase in the whole population in 2020. Actually, the incidence of new diagnoses of anxiety disorders was lower than trends observed in prior years. This is an intriguing observation, and we speculate that people more susceptible to anxiety disorders may have developed avoidant behaviors during the early phases of COVID-19, preventing them to look for medical evaluation/help, explaining the decrease in their incidence. Previous studies showed an increase in stress-related symptoms, especially anxiety and depressive symptoms, in the whole population during the COVID-19 pandemic in 2020 [11, 27]. Given the COVID-19-related socioeconomic impact, an increase in psychiatric diagnosis incident rate was anticipated, as demonstrated in other studies on prevalence since the early phases of the pandemic [7, 28]. Systematic reviews of the extant literature confirmed high numbers of mental health problems and psychosocial consequences across countries, but a wide variability in the prevalence associated with country/region preparedness to the pandemic, healthcare inequalities, and economic vulnerability indices [28–30]. Recently, a more differentiated picture of the mental health consequences according to distinct pandemic characteristics has been acknowledged. For instance, a meta-analysis showed that symptoms of anxiety (standardized mean difference [SMD] 0.40; 95% CI 0.15–0.65) and depression (SMD 0.67; 95% CI 0.07–1.27) were increased during the early phase of the pandemic compared with pre-pandemic conditions in the general population, but not in patients with COVID-19 or healthcare workers [9]. Healthcare workers experienced fluctuating course of anxiety and depressive symptoms in the early phase of the COVID-19 pandemic, indicating different impact among specific population groups [31, 32]. With the progression of the pandemic, these trends may have changed, and a picture from a late phase could be different.

In addition to addressing a later phase of the pandemic, our study focused on psychiatric diagnoses instead of psychiatric symptoms. These differences may explain the apparent contradiction with former studies. A psychiatric disorder is defined by a constellation of behavioral and cognitive symptoms that cause functional and social impairment. The clinical meaning of subthreshold psychiatric symptoms can differ from a psychiatric disorder, with the latter being typically more severe and detrimental to people’s lives, leading to a medical diagnosis [33].

Our results showed that people who experienced COVID-19 in 2020 manifested higher rates of new psychiatric disorders in 2020 than non-infected people, controlling for key demographic and substance use variables, suggesting that COVID-19 infection was directly associated with a diagnosis of new psychiatric disorders. In line with our results, two independent analyses using a large North American EHR database showed that survivors of COVID-19 had increased incidence of psychiatric disorders, mainly anxiety and psychotic disorders, compared to people with other acute medical ailments (e.g. another respiratory tract infection) [21, 34]. A more recent study using data from the Veterans Health Administration showed that post-COVID-19 patients had an increased risk of incident anxiety (HR: 1.35, CI: 1.30, 1.39; risk difference: 11.06, CI: 9.64, 12.53) and depressive disorders (HR: 1.39, CI: 1.34, 1.43; risk difference: 15.12, CI: 13.38, 16.91) compared to people without evidence of COVID-19 infection [35]. Interestingly, psychotic disorders showed the largest change in incidence rates in those with COVID-19 in both the present study and in Taquet et al. [21]. While all psychiatric disorders are linked to stress, this pathophysiological connection is stronger for anxiety/depressive disorders than for psychotic disorders, where mechanisms affecting the brain structure and function seem to play a major role [36]. This might suggest that mechanisms elicited by SARS-CoV-2 infection, e.g., vascular dysfunction and neuroinflammation, could contribute to the development of new (or exacerbation of ongoing) mental/behavioral health symptoms, subsequently contributing to a diagnosis of psychiatric disorders. It is interesting to notice that persisting symptoms following COVID-19, including anxiety, depression, sleep and cognitive (attention and executive) problems, have been increasingly recognized in the ‘long haulers’ [37].

Of notable clinical importance in the present results is the observation of a relationship between the severity of COVID-19 and the likelihood of developing a newly diagnosed psychiatric disorder. While not perfectly linear, there was a marked increase in odds of developing a psychiatric disorder at higher severity levels, when more complex medical support is required, including supplemental oxygen. In line with our findings, the above mentioned Veterans Health Administration study showed that the risks of examined outcomes (e.g. incident anxiety and depressive disorders) were highest among those who were admitted to hospital during the acute phase of COVID-19 (35). Altogether these observations may suggest that the infection and its related biological (e.g. hypoxemia, inflammatory response, hypovolemia etc.) and psychological complications (e.g. traumatic experience) contribute to long-term behavioral and cognitive symptoms or impairment.

While the current study has strengths including its sample size, comparisons of rates of previously undocumented psychiatric diagnoses across different years, inclusion of factors influencing COVID-19 severity (age, gender, race/ethnicity, medical comorbidities, tobacco and alcohol use), and the use of an instrument specifically designed to measure COVID-19 severity in claims-data, limitations must be acknowledged. Some of these limitations are inherent to a claims dataset, such as coding errors or omissions, misclassification bias, non-systematic evaluation of specific conditions (including psychiatric disorders), lack of clinical values and limited information on lifestyle and socioeconomic variables [21, 38]. In the latter instance, information on employment status, family/social support, among other factors is known to moderate stress response and psychopathology [39]. This could provide a more nuanced picture on the emergence of new psychiatric diagnoses not captured in the current dataset. Therefore, it is of paramount importance that knowledge generated in claims data is confirmed by longitudinal studies involving comprehensive neuropsychiatric and psychosocial assessments alongside biological measures, and documenting baseline or pre-existing psychiatric symptomatology. Other limitations include the focus on certain psychiatric disorders and not considering substance used disorders, eating disorders, PTSD, among others, which are more complicated to be captured in the EHR, lack of control for COVID-19 geographic distribution and temporal dynamics (i.e. its progressive spread across different North American regions, related temporal changes in therapeutic practices), not accounting for the time to COVID-19 diagnosis (thus, the exposure time was not necessarily comparable between those with COVID-19 versus those without COVID-19), and generalizability of the experience observed in the United States to other countries. Notably, the claims database is disproportionally white and, via capture of individuals who are insured in the Optum dataset, is perhaps disproportionally representative of higher or at least more stable socioeconomic levels. It is possible that the outcomes observed here may not hold in more disenfranchised factions, i.e., uninsured minority groups. While these groups are a much smaller and less representative proportion of the general US population, they may be of even greater clinical vulnerability.

In conclusion, our study did not find an increase in the trend of newly diagnosed psychiatric disorders compared to previous years in the general population. Nevertheless, people with COVID-19, especially those with more severe disease, were more likely to present with a new psychiatric disorder. The data indicate there may be utility in using medical database information to assist providers in the identification and screening of patients at increased risk for clinically significant psychiatric conditions during the ongoing period of COVID-19 in the United States.

## Supporting information

S1 Appendix (DOCX)

S1 Table Demographics by year. (DOCX)
