# Supplementary material for: Incidence rate of psychiatric disorders in 2020: The pivotal role played by SARS-CoV-2 infection
Source: PLoS One. 2022 Sep 22;17(9):e0274330. doi: 10.1371/journal.pone.0274330 (PMC9498971; doi:10.1371/journal.pone.0274330)
Supplement: S1 Appendix — (DOCX) [file pone.0274330.s001.docx]

**Appendix:**

Scale of severity of COVID-19 for use with claims data

- Severity Level 1: Person had unconfirmed report of COVID-19 during year or had post-acute symptoms after unconfirmed COVID-19.
- Severity Level 2: Person had documented COVID-19 but was asymptomatic and remained ambulatory.
- Severity Level 3: Person had documented COVID-19 with symptoms but remained ambulatory.
- Severity Level 4: Person had documented COVID-19 and received treatment at an Emergency Department but no inpatient admission.
- Severity Level 5: Person had documented COVID-19 and was admitted to an inpatient facility but did not require oxygen or advanced treatment.
- Severity Level 6: Person had documented COVID-19, was admitted to an inpatient facility, and received non-invasive oxygen.
- Severity Level 7: Person had documented COVID-19 was admitted to an inpatient facility, and received mechanical ventilation.
- Severity Level 8: Person had documented COVID-19 was admitted to an inpatient facility, and received mechanical ventilation along with Renal Dialysis or ECMO (extracorporeal membrane oxygenation).
- Severity Level 9: Person had documented COVID-19 was admitted to an inpatient facility, and died during admission.
