# Supplementary material for: Incidence rate of psychiatric disorders in 2020: The pivotal role played by SARS-CoV-2 infection
Source: PLoS One. 2022 Sep 22;17(9):e0274330. doi: 10.1371/journal.pone.0274330 (PMC9498971; doi:10.1371/journal.pone.0274330)
Supplement: S1 Table — (DOCX) [file pone.0274330.s002.docx]

| **S1 Table: Demographics by Year** | | | | | | |
| --- | --- | --- | --- | --- | --- | --- |
|  | **2018** |  | **2019** |  | **2020** |  |
| **Mean Age (SD)** | 51.68 | (24.02) | 52.41 | (23.98) | 52.83 | (23.98) |
| **Age Group** | **n (%)** | **95% CI** | **n (%)** | **95% CI** | **n (%)** | **95% CI** |
| **0-19** | 1467541 (13.84) | (13.82, 13.86) | 1431909 (13.36) | (13.34, 13.38) | 1372567 (13.12) | (13.10, 13.14) |
| **20-34** | 1338993 (12.63) | (12.61, 12.65) | 1313104 (12.25) | (12.23, 12.27) | 1264332 (12.08) | (12.06, 12.10) |
| **35-44** | 1119449 (10.56) | (10.54, 10.58) | 1104954 (10.31) | (10.29, 10.33) | 1069927 (10.23) | (10.21, 10.24) |
| **45-54** | 1231947 (11.62) | (11.60, 11.64) | 1198207 (11.18) | (11.16, 11.20) | 1129991 (10.80) | (10.78, 10.82) |
| **55-64** | 1375229 (12.97) | (12.95, 12.99) | 1367887 (12.76) | (12.74, 12.78) | 1294884 (12.38) | (12.36, 12.40) |
| **65-74** | 2028773 (19.14) | (19.11, 19.16) | 2150239 (20.06) | (20.04, 20.09) | 2194758 (20.98) | (20.95, 21.00) |
| **75 +** | 2038683 (19.23) | (19.21, 19.26) | 2151151 (20.07) | (20.05, 20.10) | 2137213 (20.43) | (20.40, 20.45) |
| **Female** | 5491262 (51.80) | (51.77, 51.83) | 5588996 (52.15) | (52.12, 52.18) | 5490136 (52.47) | (52.44, 52.50) |
| **Male** | 5109353 (48.20) | (48.17, 48.23) | 5128455 (47.85) | (47.82, 47.88) | 4973536 (47.53) | (47.50, 47.56) |
| **White** | 7180489 (70.73) | (70.70, 70.76) | 7140252 (71.05) | (71.02, 71.08) | 6766614 (71.40) | (71.37, 71.43) |
| **Black** | 1057280 (10.41) | (10.40, 10.43) | 1050586 (10.45) | (10.44, 10.47) | 972719 (10.26) | (10.24, 10.28) |
| **Asian** | 558198 (5.50) | (5.48, 5.51) | 535594 (5.33) | (5.31, 5.34) | 496991 (5.24) | (5.23, 5.26) |
| **Hispanic** | 1356400 (13.36) | (13.34, 13.38) | 1323190 (13.17) | (13.15, 13.19) | 1240683 (13.09) | (13.07, 13.11) |
| **No Comorbidity** | 3900900 (36.80) | (36.77, 36.83) | 3722083 (34.73) | (34.70, 34.76) | 3592914 (34.34) | (34.31, 34.37) |
| **1 or more comorbidity** | 6699715 (63.20) | (63.17, 63.23) | 6995368 (65.27) | (65.24, 65.30) | 6870758 (65.66) | (65.63, 65.69) |
| **No alcohol use** | 10286012 (97.03) | (97.02, 97.04) | 10432033 (97.34) | (97.33, 97.35) | 10234648 (97.81) | (97.80, 97.82) |
| **Alcohol Use** | 314603 (2.97) | (2.96, 2.98) | 285418 (2.66) | (2.65, 2.67) | 229024 (2.19) | (2.18, 2.20) |
| **No nicotine use** | 9551097 (90.10) | (90.08, 90.12) | 9727130 (90.76) | (90.74, 90.78) | 9616804 (91.91) | (91.89, 91.92) |
| **Nicotine Use** | 1049528 (9.90) | (9.88, 9.92) | 990321 (9.24) | (9.22, 9.26) | 846868 (8.09) | (8.08, 8.11) |
| **Anxiety** | 286510 (2.70) | (2.69, 2.71) | 303227 (2.83) | (2.82, 2.84) | 301504 (2.88) | (2.87, 2.89) |
| **Mood Disorders** | 318605 (3.00) | (3.00, 3.02) | 335172 (3.13) | (3.12, 3.14) | 301853 (2.88) | (2.87, 2.89) |
| **Schizophrenia** | 12226 (0.12) | (0.11, 0.12) | 12353 (0.12) | (0.11, 0.12) | 10875 (0.10) | (0.00, 0.10) |
| **OCD** | 3961 (0.04) | (0.04, 0.04) | 4326 (0.04) | (0.04, 0.04) | 4369 (0.04) | (0.04, 0.04) |

All chi-square p-values<0.05
